# The oncogenic microRNA miR-222 promotes human LINE-1 retrotransposition

**DOI:** 10.1080/15476286.2025.2511318

**Published:** 2025-05-27

**Authors:** Tomer Friehmann, Yamama Abu Mohsen, Yehuda Schlesinger, Lucy Ghantous, Lika Gamaev, Chavah Landau Zenilman, Avi Harazi, Eithan Galun, Daniel S. Goldenberg

**Affiliations:** The Goldyne Savad Institute of Gene and Cell Therapy, Hadassah University Medical Center (Hadassah Hebrew University Hospital), Jerusalem, Israel

**Keywords:** microRNA, retrotransposon, HCC, miR-222, let-7

## Abstract

The Long Interspersed Element-1 (LINE-1) contributes significantly to carcinogenesis and to tumour heterogeneity in many cancer types, including hepatocellular carcinoma (HCC), by its autonomous retrotransposition (RTP) and by its ability to retrotranspose some non-autonomous transposable elements. Previously, multiple proteins and a few microRNAs (miRs) were described as regulators of LINE-1 RTP. Here, we demonstrate that miR-222, which is oncogenic in HCC, promotes LINE-1 RTP in human HCC and some other cell lines *in vitro*, and that both miR-222-3p and miR-222-5p activate LINE-1 RTP in a cell-type specific manner. We generated miR-222-knockout mutants of the Huh7 and FLC4 hCC cell lines, and performed RNA-seq analysis of Huh7/miR-222-knockout cells and global proteomics analysis of both Huh7 and FLC4 miR-222-knockout mutants. We demonstrate that miR-222 decreases let-7c expression in both Huh7 and FLC4 cells, and that this decrease contributes to promotion of LINE-1 RTP by miR-222 in Huh7 cells.

## Introduction

Hepatocellular carcinoma (HCC) is the most common primary liver cancer and is one of the leading causes of cancer-related death worldwide [1]. Intratumor heterogeneity is frequent in HCC [2,3] and poses severe challenges for molecular targeted therapies. Heterogeneity of HCC tumours is associated with worse patient’s survival, and T cells from highly heterogeneous liver tumours have reduced cytolytic activity [4]. Genomic Transposable Elements (TEs) constitute almost half of the human genome and contribute significantly to tumour heterogeneity on both genomic and epigenomic levels [5]. Whereas most TEs in the genome are mutated and not mobile, the Long Interspersed Element 1 (LINE-1) is the only TE able to undergo autonomous retrotransposition (RTP), as well as to induce mobilization and RTP of some non-autonomous TEs [5]. Multiple copies of LINE-1 comprise about 17% of the human genome; however, most of these copies are truncated and inactive, while about 100 LINE-1 copies per each individual are full-length and active [6]. RTP events caused by LINE-1 activity result in various genetic and epigenetic mutations, including deletions, chromosomal rearrangements, changing expression of adjacent genes and generation of chimeric transcripts. In addition, LINE-1-encoded proteins also have pro-oncogenic potential [5,7]. At least a third of almost 3,000 analysed cancer genomes of 31 different cancer types, mainly of epithelial origin, including HCC and cholangiocarcinoma, had at least one LINE-1 RTP event [8]. Specifically, in HCC development, LINE-1 RTP is considered as one of drivers of hepatocarcinogenesis [9,10]. Due to a high mutagenic potential of the functional LINE-1, its mobilization and RTP in somatic cells is tightly regulated by DNA methylation and chromatin structure of appropriate genomic regions [11]; multiple cellular proteins are involved in the control of LINE-1 activity [12,13].

We aimed to determine which microRNAs (miRs) participate in regulation of LINE-1 mobilization and RTP in hepatocarcinogenesis. MiRs are widely involved in the regulation of HCC development at different stages of the disease [14,15]. Key proteins regulating processing of miRs namely, Dicer and the Microprocessor (Drosha-DGCR8 complex), suppress LINE-1 activity [16,17]. In addition, there are several known examples of miRs that control LINE-1 RTP: miR-128 and let-7 repress RTP [18,19], while miR-20, miR-16-5p, miR-153-3p, miR-30-5p and miR-138-5p activate it [20,21]. Here we demonstrate, by using the LINE-1 RTP assay (RTA) [22] and different methods of loss or gain of function of the endogenous miRs, that miR-222 (which is pro-oncogenic in HCC) promotes LINE-1 RTP in several human HCC cell lines, as well as in non-HCC cell lines, *in vitro*.

## Results

### Supervised analysis of miRs regulating LINE-1 RTP in HCC cells

**Figure 1.**
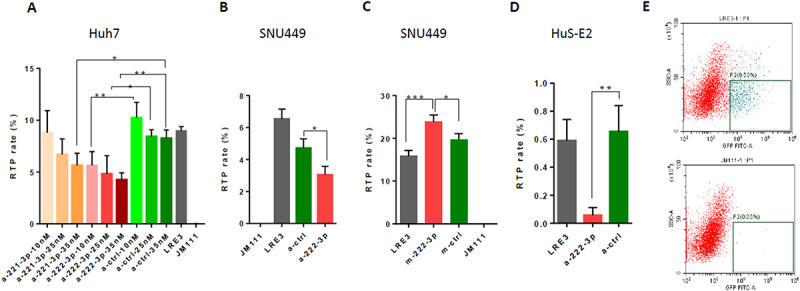
MiR-222-3p stimulates LINE-1 retrotransposition (RTP) in human HCC cells. (A) Retrotransposition assay (RTA) in Huh7 cells using LRE3-EGFP vector alone or together with increasing doses of antago-miRs (a-miRs): a-miR-222-3p, a-miR-221-3p, or control a-ctrl (JM111, a retrotransposition-defective mutant of LRE3, serves as a negative control). (B, C) RTA in SNU449 HCC cells using LRE3 vector alone or together either with a-miR-222-3p, or control a-ctrl (B), or mimic-miR (m-miR) m-miR-222-3p, or mimic-control m-ctrl (C) (40 nM all). (D) RTA in HuS-E2 cells using LRE3 vector alone or together with either a-miR-222-3p, or control a-ctrl (40 nM). (E) Representative images from FACS analysis of Huh7 cells following RTA using either retrotransposition-proficient LRE3-EGFP vector (upper panel, 9.5% GFP+ cells), or retrotransposition-deficient JM111-EGFP vector (bottom panel, 0.05% GFP+ cells). *p < 0.05, **p < 0.005, ***p < 0.0005.

To identify miRs that participate in regulation of LINE-1 RTP in HCC, we generated two lists of miRs: 1) those that were shown to be significantly differentially expressed in HCC and/or affect HCC development (based on analysis of the literature [14,23,24]), and 2) those that could target known protein regulators of LINE-1 RTP (based on our bioinformatic analysis and on analysis of the literature data [11,25]). To those miRs that were present in both these lists, we applied additional filters: targeting the maximal number of known protein regulators of LINE-1 RTP, and known expression levels of these miRs in the studied HCC cell lines [26]. By this approach, we selected the most preferable candidates: miRs 221-3p and 222-3p that share the same seed sequence and are encoded by the same intron of lncRNA MIR222HG. Both these miRs are oncogenic in HCC, based on the literature [27] and on the Kaplan–Meier plots of the 372 HCC patients (Supplementary Figure S1A,B) [28]. To evaluate the effects of candidate miRs on LINE-1 RTP in vitro, we used cell culture-based RTA that is based on comparison of RTP efficiencies of two LINE-1 vectors: LRE3 (RTP-competent) and JM111 (RTP-defective LRE3 mutant; see Figure 1(E)). Both vectors contain the EGFP reporter, which is expressed only following RTP of the LINE-1 vector into the host cell’s genome [22,29]. RTA in the Huh7 cell line with antago-miRs to these miRs demonstrated that antago-miR-222-3p (a-miR-222-3p) efficiently decreased LRE3 RTP rate at all tested concentrations, while antago-miR-221-3p was effective only at the highest tested concentration (Figure 1(A)). Antago-miR-222-3p efficiently decreased LRE3 RTP rate also in HCC SNU449 cells (Figure 1(B)) and in immortalized human foetal liver hepatocyte cell line HuS-E2 (Figure 1(D)), but not in the FLC4 cells (data not shown). Antago-miR-221-3p at concentration 40 nM did not affect LRE3 RTP rate in FLC4 and SNU449 cells (data not shown). In a complimentary experiment, addition of mimic-miR-222-3p efficiently increased LRE3 RTP rate in SNU449 cells (Figure 1(C)).

**Figure 2.**
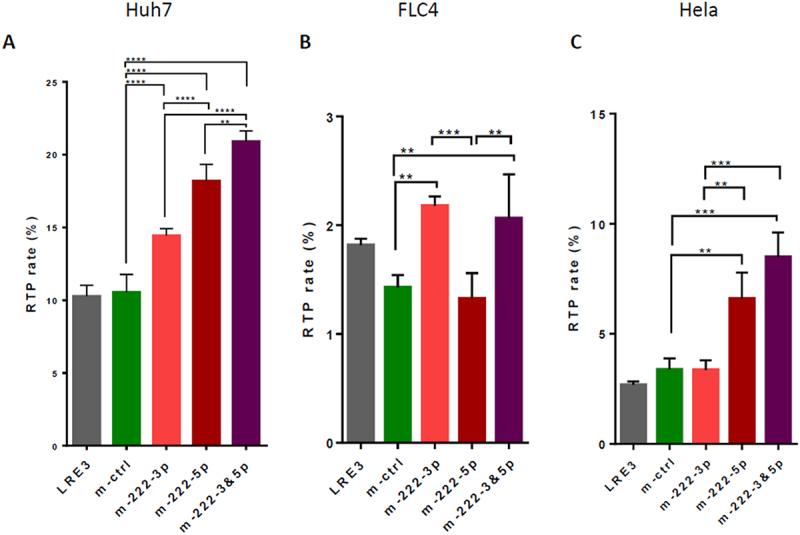
MiR-222-5p stimulates LINE-1 RTP in different cell lines. RTA in different cells using LRE3-EGFP vector alone or together with 40 nM of mimic-miRs (m-miRs): m-miR-222-3p, m-miR-222-5p, or both m-miR-222-3p and m-miR-222-5p (20 nM each; 40 nM total), or control m-ctrl. At the second day after first transfection, m-miRs were transfected repeatedly, as described (40 nM total). (A) Huh7, (B) FLC4, (C) Hela cells. *p < 0.05, **p < 0.005, ***p < 0.0005, ****p < 0.0001.

Next, we tested whether mimic-miR-222-5p may activate LINE-1 RTP, and whether it has an additive effect together with mimic-miR-222-3p. We found that mimic-miR-222-5p significantly activated LINE-1 RTP in the Huh7 and HeLa cells (Figure 2(A,C), respectively), but not in the FLC4 cells, where mimic-miR-222-3p significantly activated RTP (Figure 2(B)). Mimics miR-222-3p and miR-222-5p had an additive effect in the Huh7 (Figure 2(A)) and HeLa (Figure 2(C)) cells (despite the fact that mimic-miR-222-3p alone had no effect on RTP in HeLa cells).

**Figure 3.**
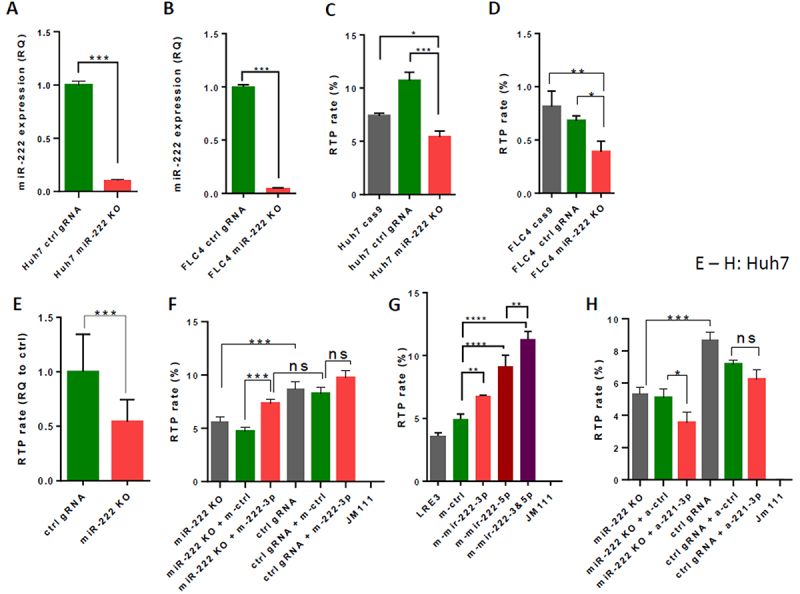
Loss of miR-222 decreases and gain of miR-222 function rescues LINE-1 RTP in HCC cells. (A, B) A significant decrease of the miR-222-3p level in the bulks of Huh7 (A) and FLC4 (B) miR-222-KO cells. (C, D) A significant decrease of LINE-1 RTP in the bulks of Huh7 (C) and FLC4 (D) miR-222-KO cells. Controls (also bulks) – the same Huh7 and FLC4 Cas9-expressing clones transduced with a lenti-vector expressing a control gRNA (ctrl gRNA). (E) RTA in Huh7 Cas9-expressing clones transduced with a lenti-vector expressing either a control gRNA (ctrl gRNA), or gRNA for miR-222: summary of 3 repetitive experiments using 4 single cell clones per group (miR-222-KO and controls). (F) LINE-1 RTA rescue experiment in single clones of Huh7 cells (one – miR-222-KO and one – control gRNA) transfected with mimic-RNAs – either m-miR-222-3p, or m-ctrl (20 nM). (G) LINE-1 RTA rescue experiment in Huh7/miR-222-KO cells (bulks). Concentration of mimics – as in Figure 2. (H) LINE-1 RTA experiment in single clones of Huh7 cells (one – miR-222-KO and one – expressing a control gRNA, same clones as in (F) transfected with antago-RNAs – either a-miR-221-3p, or a-ctrl (20 nM). *p < 0.05, **p < 0.005, ***p < 0.0005, ****p < 0.0001.

To explore further the effect of miR-222 on LINE-1 RTP, we performed knockout (KO) of miR-222 in Huh7 and FLC4 cells using the CRISPR-Cas9 system. First, we generated Huh7 and FLC4 clones stably expressing Cas9. The resulting single clones were tested for their efficiency to produce KO of the CD47 gene upon transduction with a lenti-vector expressing gRNA targeting CD47, and the most efficient in this test clones were chosen for further work (Supplementary Figure S2A-D). Then, miR-222-KO derivatives of these clones were generated by transduction with a lenti-vector expressing a gRNA targeting miR-222 genomic sequence. In the bulks of both generated miR-222-KO HCC cell lines, miR-222 levels (Figure 3(A,B)) and LINE-1 RTP (Figure 3(C,D)) were significantly reduced. In order to check whether the effect of miR-222-KO mutation on LINE-1 RTP could be caused by its effect on cell proliferation, we compared proliferation rates of Huh7/Cas9 and FLC4/Cas9 cells transfected with lenti-vector expressing either gRNA targeting miR-222, or a control gRNA – and demonstrated that miR-222-KO mutation in both these cell lines did not affect cell proliferation rates (Suppl. Fig. S3A,B). Due to co-localization of miR-222 and miR-221 in the same genomic locus, we checked whether loss of miR-222 affects expression of the neighbouring miR-221. Expression levels of both miR-221-3p and miR-221-5p were decreased about 40% in bulks of Huh7/miR-222-KO mutants, while they were not significantly changed in bulks of FLC4/miR-222-KO mutants (Suppl. Fig. S4A,B, respectively; melting curves in Suppl. Fig. S4C-F demonstrate that the quantified PCR-product for both arms of miR-222 was irrelevant, whereas for miR-221 - relevant). In order to explore the molecular mechanisms responsible for the decrease of LINE-1 RTP in the absence of miR-222, we have grown individual clones of the Huh7/Cas9 cells transduced with a lenti-vector expressing either a gRNA to miR-222, or a control gRNA that has no known targets in the human genome (4 clones in each group). LINE-1 RTP in the Huh7/Cas9/miR-222-KO clones was significantly reduced compared to control Huh7/Cas9/ctrl-gRNA clones (Figure 3(E)). The ‘rescue’ RTA with LINE-1 and mimic-miR-222-3p (or mimic-control) transfected into Huh7 stable single clones (one – miR-222-KO and one – expressing control gRNA) demonstrated that mimic-miR-222-3p at a low concentration (20 nM) may provide rescue to LINE-1 RTP in the miR-222-KO cells, but not in the cells stably expressing control gRNA (Figure 3(F)). The rescue RTA with LINE-1 and both mimics m-miR-222-3p and m-miR-222-5p (or mimic-control) transfected into bulk of Huh7/miR-222-KO and Huh7/ctrl-gRNA cells, demonstrated that each mimic rescues LINE-1 RTP in the Huh7/miR-222-KO cells, and that together they have an additive effect (Figure 3(G)). RTA with LINE-1 and antago-miR-221-3p (or antago-control) at a low concentration (20 nM) transfected into Huh7 stable single miR-222-KO clones (the same clones as in Figure 3(F)) demonstrated that antago-miR-221-3p significantly decreased LINE-1 RTP in the Huh7/miR-222-KO cells, but not in the Huh7 cells stably expressing control gRNA (Figure 3(H)). These results imply that both miR-222 and miR-221-3p contribute to activation of LINE-1 RTP in Huh7 cells.

### Transcriptomic analysis of Huh7 miR-222-KO clones

**Figure 4.**
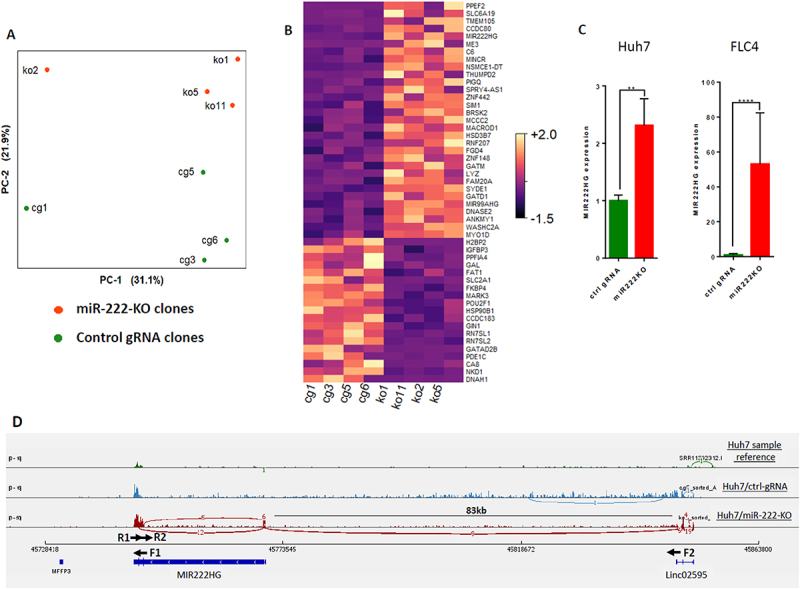
RNA-seq analysis of Huh7/miR-222-KO clones. (A) Principal component analysis (PCA) of RNA-seq data of four miR-222-KO (ko) and four control (cg) Huh7 clones. (B) Heat map of the RNA-seq results showing 50 most differentially expressed genes between groups. (C) Increased expression of the MIR222HG transcript in stable miR-222-KO mutants versus stable cells expressing control gRNA of the Huh7 (left panel; bulks, *n* = 3) and FLC4 (right panel; 3 single cell clones in each group; *n* = 9) cell lines (RT-PCR with primers F1 & R1). (D) Sashimi plot of representative Huh7 (SRR11392312), and Huh7 derivatives harbouring either control gRNA, or miR-222 KO (top to bottom, respectively). The plot shows RNA-seq reads aligned to the human genome and revealing existing splicing junctions. Black arrows show primers used for RT-PCR. **p < 0.005, ****p < 0.0001.

In order to reveal the molecular mechanisms responsible for the decrease of LINE-1 RTP in the absence of miR-222, we analysed transcriptomes of the stable single clones Huh7/Cas9/miR-222-KO versus similar control clones expressing a control gRNA that has no targets in human genome (four clones in each group). Principal Component Analysis of the RNA-seq results demonstrated that knockout samples are well separated from the control samples (Figure 4(A)). Heatmap of the 50 most differentially expressed genes between two groups (Figure 4(B)) also demonstrates a separation between groups, as well as a high variability of gene expression levels by individual samples inside each group: only 23 genes differentially expressed between two groups had adjusted p-value <0.05. Gene Set Enrichment Analysis of RNA-seq data (Suppl. Fig. S5) revealed that loss of miR-222 converts Huh7 cells to less cancerous and more hepatocyte-like cells by decrease of several pro-cancerous processes (DNA replication, mitosis, mRNA processing, regulation of apoptosis) and increase of normal hepatocyte functions (translation of proteins, import of peroxisomal proteins, complement and coagulation cascades). Loss of miR-222 in Huh7 cells resulted also in increased expression of genes associated with survival of liver cancer patients and genes, whose down-regulation was associated with liver cancer recurrence (Suppl. Fig. S5, upper panel). Importantly, loss of miR-222 resulted also in down-regulation of the NIK (MAP3K14) – non-canonical NF-kB signalling that mediates expression of HCC driver NRF2 (NFE2L2) [30,31]. Remarkably, expression of both RN7SL1 and RN7SL2 genes encoding the pro-oncogenic 7SL lncRNA [32,33] was significantly decreased in Huh7/miR-222-KO cells (Figure 4(B)).

**Figure 5.**
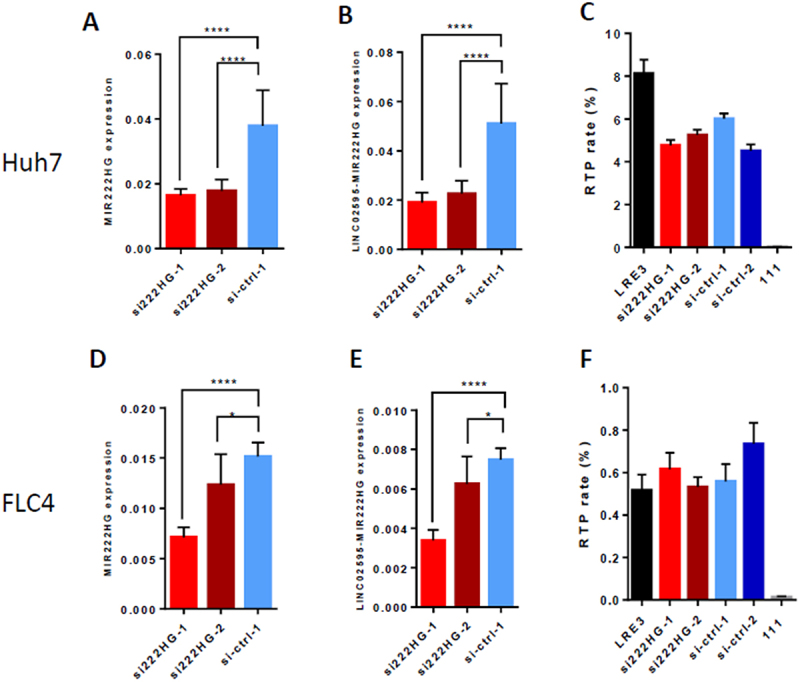
Knockdown of the MIR222HG does not affect efficiency of LINE-1 RTP. Knockdown of the MIR222HG using two pairs of siRNAs in bulks of stable Huh7 (A – C) and FLC4 (D – F) miR-222-KO mutants. (A, B) Both pairs of siRNAs designed against the last exon of MIR222HG efficiently decrease levels of the MIR222HG transcript ((A), detected by RT-PCR using primers F1, R1) and the Linc02595-MIR222HG transcript ((B), detected by RT-PCR using primers F2, R2) in Huh7/miR-222-KO cells at the second day following transfection. (C) Neither pair of siRNAs against MIR222HG affects LINE-1 RTP in Huh7/miR-222-KO cells. (D, E) SiRNA pairs si222HG–1 (efficiently) and si222HG–2 (less efficiently) decrease the levels of MIR222HG transcript ((D), detected by RT-PCR using primers F1, R1) and Linc02595-MIR222HG transcript ((E), detected by RT-PCR using primers F2, R2) in FLC4/miR-222-KO cells at the second day following transfection. (F) neither pair of siRNAs against MIR222HG affects LINE-1 RTP in FLC4/miR-222-KO cells. LINE-1 RTP was tested using LRE3-GFP vector and two different control siRNAs (si-ctrl-1 and si-ctrl-2). Gene expression in A, B and D, E was normalized to that of EEF2. *p < 0.05, ****p < 0.0001.

One of the most significantly upregulated transcripts in Huh7/miR-222-KO mutant clones was lncRNA MIR222HG, encoding both miR-221 and miR-222 (Figure 4(B)). We confirmed its significant upregulation in miR-222-KO mutants of both Huh7 and FLC4 cells by RT-PCR (Figure 4(C) – cells’ bulks, Suppl. Fig. S6A – single Huh7 clones). The known variants of the lncRNA MIR222HG have variable length and are produced by splicing of few exons located in the interval of about 20 kb in human X chromosome (Suppl. Fig. S6B; see also [34,35]). According to Ensembl database, MIR222HG has two exons, while group of K.V. Prasanth identified in it four exons [34]. In our Huh7/miR-222-KO cells, we could not identify their first exon either by RNA-seq, or by PCR (data not shown), but we detected their ‘exon 3’ which is absent in the Ensembl data. The MIR222HG transcript which we have detected in the Huh7/miR-222-KO cells, consists of three exons: exon 1 (that is identical to exon 1 of Ensembl and to exon 2 of Prasanth’s team), exon 2 (that is identical to exon 3 of Prasanth’s team and is absent in Ensembl), and exon 3 (that is identical to exon 4 of Prasanth’s team and to exon 2 in Ensembl) (Suppl. Fig. S6E, F). Remarkably, in the control Huh7 cells, we could detect only exon 3 of MIR222HG (Figure 4(D)). We have also found that in miR-222-KO cells, a new variant of MIR222HG transcript, which is produced by splicing with exons of a distant lncRNA Linc02595, located 83 kb apart from the end of the MIR222HG gene (Figure 4(D), Suppl. Fig. S6C). This new Linc02595-MIR222HG splicing is induced also by a knockdown of miR-222-3p in HeLa, Huh7 and FLC4 cells (Suppl. Fig. S6D – S6G). In order to test – whether upregulation of MIR222HG and/or Linc02595-MIR222HG expression contributes to the decrease of LINE-1 RTP in miR-222-KO cells – we performed RTA with LRE3 vector in the presence of siRNAs against the last (and the largest) exon of MIR222HG (two pairs of siRNAs were used, each containing two different siRNAs). Both siRNA pairs efficiently decreased the levels of both MIR222HG and Linc02595-MIR222HG transcripts in Huh7/miR-222-KO cells (Figure 5(A,B)), and one of them efficiently decreased the levels of both these transcripts in FLC4 cells as well (Figure 5(D,E)). Nevertheless, none of the siRNA pairs affected the level of LRE3 RTP in these cells (Figure 5(C,F)). These results demonstrate that: 1) miR-222 negatively regulates expression of its host gene MIR222HG, including expression of its new, alternatively spliced chimeric transcript; and 2) decrease of LINE-1 RTP in tested miR-222-KO cells is not caused by upregulation of either the known MIR222HG transcript, or the newly discovered chimeric transcript Linc02595-MIR222HG.

### The role of let-7c and miR-99a in regulation of LINE-1 RTP

**Figure 6.**
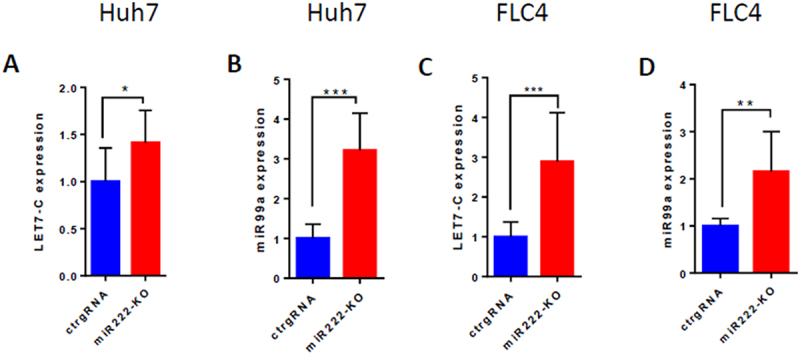
Loss of miR-222 results in upregulation of let-7c-5p and miR-99a-5p expression. Expression of miRNAs let-7c-5p (A, C) and miR-99a-5p (B, D) was measured in single cell clones isolated from either Huh7/miR-222-KO (A, B), or FLC4/miR-222-KO (C, D), or their appropriate control gRNA-expressing cells. For Huh7 cells − 4 clones in each group; for FLC4 cells − 3 clones in each group; *p < 0.05 **p < 0.005 ***p < 0.0005.

**Figure 7.**
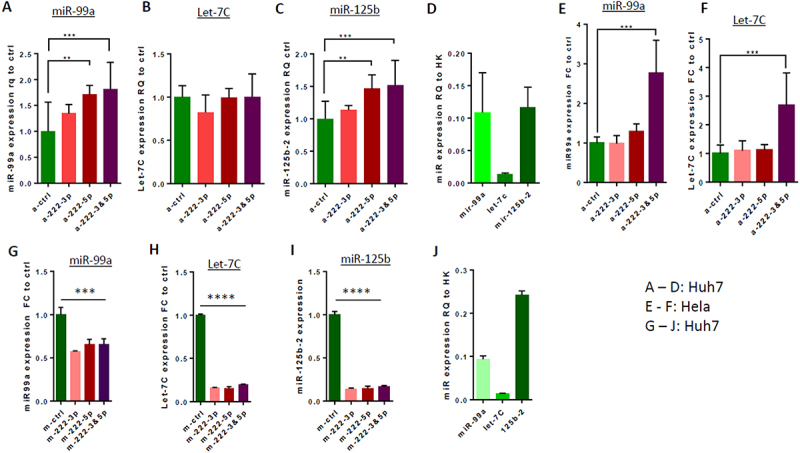
Effects of modulation of miR-222 level on expression of miR-99a-5p, let-7c-5p, and miR-125b-5p in Huh7 and HeLa cells. Expression of miR-99a-5p (A, E, G), miRNAs let-7c-5p (B, F, H), and miR-125b-5p (C, I) in bulks of Huh7 (A – D, G – J) and HeLa (E, F) cells following transfection with either antago-miRs (a-miRs, A – F), or mimic-miRs (G – J) to miR-222-3p and/or miR-222-5p. (A-C and E-F) expression of each miR of miR-99a cluster following antago-miRs transfection relative to their expression in the same cells transfected with a control antago-miR. (D) Expression of miRNAs miR-99a-5p, let-7c-5p and miR-125b-5p in Huh7 cells transfected with a control antago-miR, relative to a standard ‘housekeeping’ small RNA. (G – I) Decreased expression in Huh7 cells of miRNAs miR-99a-5p (G), let-7c-5p (H) and miR-125b-5p (I) following transfection of cells with mimics to miR-222-3p and/or miR-222-5p, relative to expression of each of these miRs in Huh7 cells transfected with a control mimic-miR. (J) Expression of miRNAs miR-99a-5p, let-7c-5p and miR-125b-5p in Huh7 cells transfected with a control mimic-miR, relative to a standard ‘housekeeping’ small RNA. Concentrations of antago-miRs and mimic-miRs: 40 nM for each, when only one type is applied; 20 nM each one for antago/mimic-miR-222-3p and antago/mimic-miR-222-5p when they are applied together (40 nM total); **p < 0.005, ***p < 0.0005, ****p < 0.0001.

**Figure 8.**
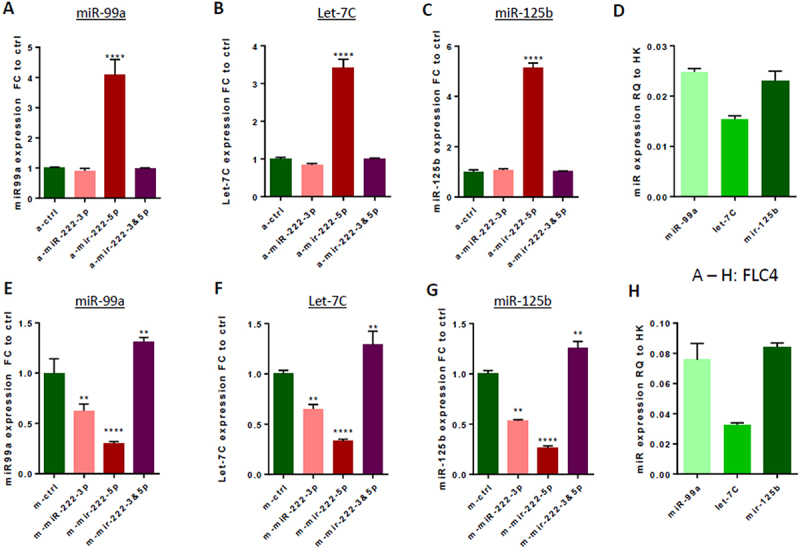
Effects of modulation of miR-222 level on expression of miR-99a-5p, let-7c-5p, and miR-125b-5p in FLC4 cells. Expression of miR-99a-5p (A, E), miRNAs let-7c-5p (B, F), and miR-125b-5p (C, G) in bulks of FLC4 cells following transfection with either antago-miRs (a-miRs, A-D), or mimic-miRs (E-H) to miR-222-3p and/or miR-222-5p. (A-C) Expression of each miR of miR-99a cluster following antago-miRs transfection relative to their expression in the same cells transfected with a control antago-miR. (D) Expression of miRNAs miR-99a-5p, let-7c-5p and miR-125b-5p in FLC4 cells transfected with a control antago-miR, relative to a ‘housekeeping’ small RNA. (E-G) Decreased expression in FLC4 cells of miRNAs miR-99a-5p, let-7c-5p and miR-125b-5p following transfection of cells with mimics to miR-222-3p and/or miR-222-5p, relative to expression of each of these miRs in FLC4 cells transfected with a control mimic-miR. (H) Expression of miRNAs miR-99a-5p, let-7c-5p and miR-125b-5p in FLC4 cells transfected with a control mimic-miR, relative to a standard ‘housekeeping’ small RNA. Concentrations of antago-miRs and mimic-miRs: 40 nM for each, when only one type is applied; 20 nM each one for antago/mimic-miR-222-3p and antago/mimic-miR-222-5p when they are applied together (40 nM total); **p < 0.005, ****p < 0.0001.

In an effort to reveal how miR-222 regulates LINE-1 RTP in HCC cells, we searched in our RNA-seq data for additional candidate miR transcripts involved in this process. We identified in the miR-222-KO clones of Huh7 cells, a modestly (about 1.5 fold) upregulated transcript – the lncRNA MIR99AHG (Figure 4(B)), which encodes miR-99a, let-7c and miR-125b-2 (Suppl. Fig. S7A). We tested expression of different miRs encoded by this transcript using a ‘loss of function’ approach for miR-222, and revealed that both miR-99a-5p and let-7c-5p were significantly overexpressed in single miR-222-KO clones of both Huh7 and FLC4 cells (Figure 6(A–D)). Knockdown of miR-222-3p and −5p isoforms by antago-miRs produced different results in different cell lines: in Huh7 cells, knockdown of miR-222-3p was not effective, while knockdown of miR-222-5p increased the levels of miR-99a-5p and miR-125b-5p (Figure 7(A–C)). In HeLa cells, expression of miR-99a-5p and let-7c-5p was significantly increased only following transfection with both antago-miR-222-3p and antago-miR-222-5p (Figure 7(E,F)). Using a complimentary, ‘gain of function’ approach, we demonstrated that each of mimic-miRs for miR-222-3p and miR-222-5p efficiently decreased expression of miR-99a-5p (about 40%), let-7c-5p (about 5-fold) and miR-125b-5p (about 5-fold) in Huh7 cells (Figure 7(G,H,I), respectively). Relative expression levels of miR-99a-5p, let-7c-5p and miR-125b-5p in Huh7 cells are shown in Figure 7(D) (following transfection with antago-control) and Figure 7(J) (following transfection with mimic-control). Similar modulations of miR-222 levels in the FLC4 cells demonstrated that only antago-miR-222-5p significantly upregulated expression of all three miRs encoded by MIR99AHG (Figure 8(A–C)), while both miR-222-3p and miR-222-5p mimics significantly decreased expression of these three miRs (mimic-miR-222-5p was more efficient; Figure 8(E–G)). Relative expression levels of miR-99a-5p, let-7c-5p and miR-125b-5p in FLC4 cells are shown in Figure 8(D) (following transfection with antago-control) and Figure 8(H) (following transfection with mimic-control). Importantly, miR-125b-2-5p encoded by the MIR99AHG is identical to miR-125b-1-5p encoded by the MIR100HG (Suppl. Fig. S7A-C); thus, expression of the miR-125b-5p in Figures 7 and 8 is actually a sum of expression levels of both these isoforms.

**Figure 9.**
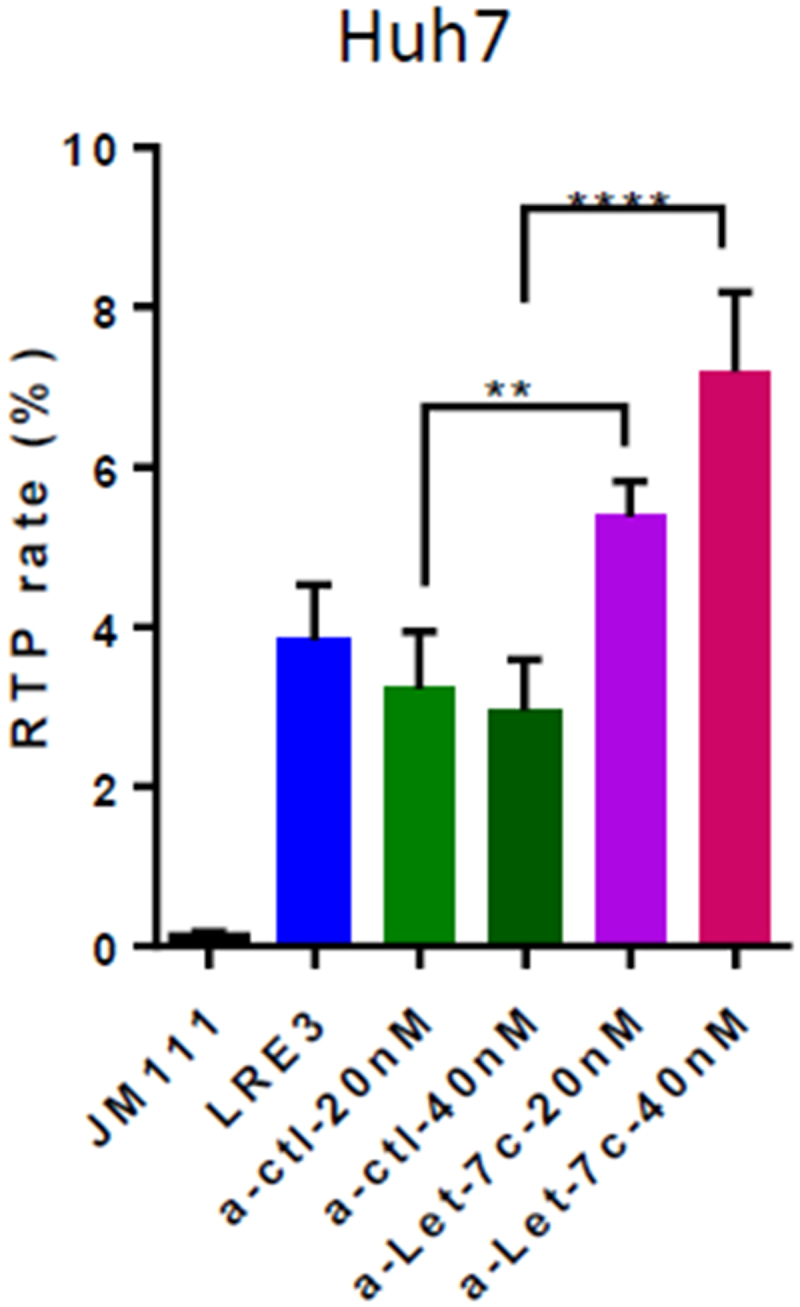
Antago-let-7c rescues LINE-1 RTP in Huh7/miR-222-KO cells in vitro. RTA in Huh7/miR-222-KO cells (bulk) using LRE3-EGFP vector alone or together with antago-miR (a-miR) to let-7c-5p, or control a-ctrl (JM111 – negative control, a retrotransposition-defective mutant of LRE3). Concentration of antago-miRs: 40 nM for a-ctrl, 20 nM and 40 nM for a-let-7c-5p; **p < 0.005, ****p < 0.0001.

In order to explore the role of miRs encoded by the MIR99AHG in LINE-1 RTP, we performed RTA using the LRE3-EGFP vector and antago-miRs to miR-99a and let-7c. Our results demonstrated that in Huh7/miR-222-KO cells, only antago-let-7c was able to increase LINE-1 RTP by dose-dependent manner (Figure 9), while antago-miR-99a was ineffective (Suppl. Fig. S8). However, in FLC4/miR-222-KO cells, neither antago-let-7c nor antago-miR-99a affected LINE-1 RTP (data not shown). Importantly, the antago-let-7c was not strictly specific, and could also decrease levels of the other let-7 family members, especially let-7a, which is highly homologous to let-7c (they differ in one nucleotide only). These our findings demonstrate that upregulation of let-7c in Huh7/miR-222-KO cells (but not in similar FLC4 mutants) contributes to the decrease of LINE-1 RTP in these cells. However, we cannot exclude contribution of other members of the let-7 family (e.g. let-7a) to this process.

### Global proteomics of the Huh7 and FLC4 miR-222-KO mutant cells

We have performed three rounds of global proteomics analyses of Huh7/miR-222-KO and FLC4/miR-222-KO cells (bulks of these mutants; in both cases, we used as controls bulks of the parental Huh7/Cas9 or FLC4/Cas9 cells and their derivatives transfected with a lentiviral vector expressing the control (cg) gRNA). A very limited number of proteins were significantly differentially expressed between the control (cg) and miR-222-KO (ko) cells of each type in all three proteomics rounds: COL2A1 protein was downregulated in Huh7 cells, while BAAT and METTL7A proteins were upregulated in FLC4 cells (Suppl. Fig. S9; fold-change threshold = 1.8). Differentially expressed proteins were mainly cell-type specific: among proteins that were up- or down-regulated in at least two proteomics rounds for each cell type, there was only one common up-regulated protein (TACO1) for Huh7 and FLC4 cells, and there was none down-regulated common protein. Even a decrease of the fold-change threshold to 1.5 did not change this cell-specific pattern of proteins’ aberrant expression. Importantly, among all proteins aberrantly expressed in at least two proteomics rounds (counted in Suppl. Fig. S9), statistically significant were only numbers of FLC4 proteins common for two rounds performed in the Israeli Technological Institute (shown in Suppl. Fig. S9 as red and blue circles): 9 up-regulated and 14 down-regulated (for both groups *p* < 0.01). Among these 23 proteins, there was only one known regulator of LINE-1 RTP: SIRT1. However, SIRT1 is a known repressor of LINE-1 RTP [36], and it was downregulated in FLC4/miR-222-KO cells; thus, its aberrant expression cannot explain the decrease of LINE-1 RTP in these cells. In contrast to bioinformatic predictions, among proteins upregulated in Huh7/miR-222-KO and FLC4/miR-222-KO cells very few were miR-222 targets. For proteins upregulated in at least two proteomics rounds, only two were predicted targets of miR-222-3p: one – in Huh7 cells (among 7 upregulated proteins) and one – in FLC4 cells (among 25 upregulated proteins). On the other hand, in both Huh7 and FLC4, many proteins that were predicted bioinformatically as targets of miR-222-3p or miR-222-5p (including proteins that were targeted by both miR-222 arms) were similarly expressed in miR-222-KO mutants and control cells. These data demonstrate that the classic targeting of proteins by miR-222 arms was not efficient in the tested Huh7 and FLC4 cells.

## Discussion

### Regulation of LINE-1 RTP by miRs

In this study, we aimed to determine miRs that regulate LINE-1 RTP in HCC. The total effect of miRs on LINE-1 is repressive, since mutations in genes encoding miRs-processing machinery result in LINE-1 activation [17]. There are several known examples of miRs that control LINE-1 RTP, and all of them use different molecular mechanisms to control this process. MiR-128 inhibits LINE-1 RTP by at least three different mechanisms: by direct binding to LINE-1 RNA resulting in its degradation [37], by down-regulating the nuclear import factor TNPO1 [38], and by down-regulating hnRNPA1 protein [39]. Let-7 represses LINE-1 RTP by direct binding to its RNA and interfering with translation of its ORF2, which is both LINE-1 reverse transcriptase and transposase [19]. Although miR-20 may bind to LINE-1 RNA at two sites, it activates LINE-1 RTP by yet unknown mechanism(s) [20]. Recently, it was demonstrated that four miRs – miR-16-5p, miR-153-3p, and (less efficiently) miR-30-5p and miR-138-5p - activate LINE-1 RTP by the same mechanism: by downregulating RTP repressor MOV10 [21]. Now, we add to this list two additional miRs that activate LINE-1 RTP: miR-222 (both −3p and −5p arms) and miR-221-3p (although less efficient than miR-222).

### Regulation of miR-221/222 expression and their roles in cancer development

MicroRNAs miR-221 and miR-222 are produced from the same intron of the lncRNA MIR222HG encoded by the human X chromosome. In most published articles, names ‘miR-221’ and ‘miR-222’ usually designate the −3p arms of these miRs, which are highly homologous and share the same seed sequence. Both miR-221 and miR-222 target many mRNAs and lncRNAs and regulate multiple processes associated with carcinogenesis, acting mainly as oncogenes, but for some cancer types they act as tumour suppressors [40,41]. In HCC, both miR-221 and miR-222 are highly upregulated and function as oncogenes, promoting different stages of hepatocarcinogenesis by multiple mechanisms [27,42,43]. The effects of these two miRs on HCC development differ in that miR-221 regulates multiple stages of hepatocarcinogenesis starting from chronic liver injury [42], whereas miR-222 is specifically upregulated in HCC compared to benign liver tumours [44]. Here, we demonstrate that microRNA miR-222 promotes LINE-1 RTP in several cell lines *in vitro*, including HCC cell lines, and that miR-221 also promotes it in Huh7 cells (although, less efficiently than miR-222). Importantly, we have found that both miR-222-3p and miR-222-5p arms promote LINE-1 RTP. According to the database of HCC cell lines generated by Caruso S. et al. [26], in both Huh7 and JHH4 (which is parental to FLC4) cell lines, level of miR-222-5p is higher than that of miR-222-3p. Thus, activation of LINE-1 RTP by both miR-222 arms and, less efficiently, by miR-221-3p, discovered in our current study, should be considered as an additional oncogenic mechanism of these miRs in HCC development (and, maybe, in other cancer types as well). Remarkably, both these miRs are increasing HCC resistance to drugs: miR-221 – to sorafenib [45,46], and miR-222 – to cisplatin [47,48]. Possible contribution of the increased LINE-1 RTP into this HCC phenotype also should not be excluded.

Similar to many other lncRNAs that encode miRs, MIR222HG has some functions in the cell, which do not depend on miRs that it encodes. e.g. in prostate cancer, increased MIR222HG expression promotes castration-resistance, and MIR222HG level only modestly correlates with miR-221/222 expression [35]. In a cell line generated from lung foetal fibroblasts, MIR222HG promotes the cell cycle re-entry post quiescence independently on miR-221/222, and miR-222 level does not depend on the level of spliced MIR222HG [34]. In cell lines produced from different cancer types, the promoters and spliced variants of the MIR222HG are different [34,35]. Now, we have demonstrated, for the first time, that in HCC cell lines, depletion of miR-222 results both in increased level of MIR222HG, and in the appearance of a new MIR222HG variant – spliced Linc02595-MIR222HG transcript (the latter phenomenon takes place also in the HeLa cells depleted of miR-222-3p). These findings imply that miR-222 negatively regulates MIR222HG expression and splicing in the studied cell lines. However, MIR222HG knockdown did not affect LINE-1 RTP in miR-222-KO mutants of Huh7 and FLC4 cells, demonstrating that the increased expression of the Cas9-mutated MIR222HG and appearance of the new spliced Linc02595-MIR222HG transcript did not cause the LINE-1 RTP decrease in these cells.

### Results of RNA-seq of Huh7/miR-222-KO mutants

In addition to upregulation of MIR222HG, the most significant and interesting RNA-seq results were downregulation of the RN7SL1/2 and upregulation of the MIR99AHG lncRNAs (Figure 4B). Both RN7SL1 and RN7SL2 genes encode the 7SL lncRNA, which is a part of the signal recognition particle, and also has multiple other functions in the cell. 7SL RNA is oncogenic and is upregulated in tumours of several cancer types, including liver cancer [49]. Downregulation of the 7SL RNA in Huh7/miR-222-KO cells contributes to the less oncogenic phenotype of these cells, in tune with the GSEA results (Suppl. Fig. S5). In accordance with upregulation of the MIR99AHG lncRNA, miRs encoded by it, miR-99a-5p and let-7c-5p, were upregulated in miR-222-KO mutants of Huh7 and FLC4 cells (Figure 6). Temporal loss of miR-222-5p and gain of either −3p or −5p arms of miR-222 caused upregulation and down-regulation, respectively, of miR-99a-5p, let-7c-5p and 125b-5p in both cell types (Figure 7 and Figure 8). These three miRs function mainly as tumour suppressors in many cancer types, including HCC [50–53] and cholangiocarcinoma [54]. Kaplan–Meier plots of these three miRs in HCC also demonstrate their tumour suppressive effect: patients with higher expression of each of these miRs in tumours have prolonged survival (Suppl. Fig. S10A-C).

Remarkably, lncRNA MIR99AHG, similar to MIR222HG and to many other lncRNAs encoding miRs, has its own activity in cells, which does not depend on miRs that it encodes: e.g. in colorectal cancer, it regulates alternative splicing, changing by this chromatin state and promoting metastasis [55]. Thus, we cannot exclude that a modest upregulation of MIR99AHG in HCC cells devoid of miR-222 may affect LINE-1 RTP by some additional ways, independent of miRs encoded by this lncRNA.

Taking into account the known molecular mechanisms by which miR-128-3p affects LINE-1 RTP [37], we tested and confirmed that loss of miR-222 in LRE3-transfected Huh7 and FLC4 cells, did not decrease the steady-state level of the LINE-1-EGFP transcript (Suppl. Fig. S11A,B), and did not increase the level of miR-128-3p in LRE3-transfected Huh7 cells (Suppl. Fig. S12).

### Protein targets of miR-222

The results of our global proteomics assays were frustrating due to a low reproducibility: among proteins differentially expressed between miR-222-KO and control cells in any single assay, only about 10% were similarly differentially expressed in two assays, and only 3 proteins were similarly differentially expressed in all 3 assays (2 upregulated in FLC4 cells and 1 downregulated in Huh7 cells; Suppl. Fig. S9). Surprisingly, very few proteins upregulated in miR-222-KO cells were defined bioinformatically as targets of any miR-222 arm. Among 7 proteins upregulated in at least 2 assays in Huh7 cells, only one, TERF2, is defined as miR-222-3p target; similarly, among 25 proteins upregulated in at least 2 assays in FLC4 cells, only one, AGFG2, is defined as miR-222-3p target. We tested expression of TERF2 by immunoblotting and could not validate its differential expression in Huh7/miR-222-KO cells (data not shown). Our proteomic assays detected about 3,500–4,000 proteins per assay – mainly, highly expressed proteins. Although we could not detect low expressed proteins that are known as miR-222 targets either bioinformatically, or experimentally, nevertheless, we could detect many such proteins – and almost all of them were similarly expressed between miR-222-KO and control cells. Remarkably, some of these similarly expressed proteins were bioinformatically defined as targets of both miR-222-3p and miR-222-5p arms. Based on these data, we conclude that the bioinformatically predicted canonical targeting of proteins by miR-222 arms was not efficient in the tested HCC cells. This could be caused, e.g. either by known extensive variation of miR-222 isoforms [56], or by 8-oxoguanine modification of miR-222 that changes miR’s targeting specificity [57].

### Possible mechanisms of LINE-1 RTP promotion by miR-222

Although, at this stage, we do not know the exact molecular mechanism(s) responsible for the effect of miR-222/221 on LINE-1 RTP, our data demonstrate that their effect on expression of several other miRs and lncRNAs may significantly contribute to LINE-1 RTP activation. The absence in our proteomics assays of the prominent protein candidates that could act as mediators of the miR-222/221 effect on LINE-1 RTP, could be explained by the findings of the C.P. Bracken’s group demonstrating that major effects of miRs’ manipulation may be transcriptional [58], and be a sum of multiple small effects of several aberrantly expressed miRs and lncRNAs – so called ‘crowd control’ [59]. Taking into account that miR-222 is specifically upregulated in the switch from benign to malignant liver tumours [44], we may suggest that activation of LINE-1 RTP by miR-222 significantly contributes to this process. Known data on LINE-1 RTP protein regulators make it possible to speculate that miR-222 may participate in such regulatory processes. E.g., the SRSF1 protein (which is an oncogene - [60]) activates LINE-1 RTP [13], and its over-expression in HeLa cells causes upregulation of miR-221 and miR-222, while down-regulation of miR-99a, miR-let-7c, miR-125a,b [61]. On the other hand, transcriptional repressor ZBTB16/PLZF represses LINE-1 RTP [62], and represses also miR-221/miR-222 cluster [63]. Thus, both the activation of LINE-1 RTP by SRSF1 and its repression by ZBTB16/PLZF could be, at least partially, attributed to the effects of these proteins on miR-221/miR-222 expression.

### Cell-type specificity of LINE-1 RTP regulation

Regulation of LINE-1 RTP by proteins may be cell-specific: e.g., genome-wide screen for such regulators in two cell lines derived from different human tissues revealed that most detected regulators were common for both cell lines, but significant sets of regulators were cell-type specific [13]. In our study, two HCC cell lines – Huh7 and FLC4 – differed significantly in global proteomics assays, demonstrating negligible numbers of common proteins affected by the loss of miR-222. There are many known differences between these cell lines, e.g.: miR-122 level is high in Huh7 while low in FLC4 (known data, validated by us experimentally; data not shown); low levels of TGF-beta & Smad7, and sensitivity to TGF-beta-induced cytostasis in Huh7, while high levels of TGF-beta & Smad7, and resistance to TGF-beta-induced cytostasis in FLC4 [64]; different spectrum of proteins induced by UVC-irradiated apoptotic FLC4 cells compared with other tested HCC cell lines, including Huh7 [65]. Nevertheless, we found that miR-222 activates LINE-1 RTP in three HCC cell lines (Huh7, FLC4 and SNU449), in non-cancer immortalized human hepatocyte (HuS-E2) and in HeLa cells. Thus, miR-222 activates LINE-1 RTP by some mechanism that is common for these cell lines and, most probably, involves let-7c, but does not involve proteins that were detected by our proteomics assays. We acknowledge that our findings are limited by *in vitro* cell culture models and should be validated *in vivo*. Further studies should reveal the exact molecular mechanism(s) by which miR-222 activates LINE-1 RTP and thus contributes to the development of HCC malignancy.

## Methods

### Cell cultures

All cells are from the cell collection of our institute. The human HCC cell lines Huh7 and FLC4, along with the cervical cancer cell line HeLa, were cultured in DMEM, while HCC SNU449 cells – in RPMI medium, supplemented with 10% foetal calf serum and 1% penicillin/streptomycin (Thermo Fisher Scientific, Waltham, MA, USA). Immortalized human hepatocytes HuS-E2 [66] were grown as described in [67]. All cells were incubated at 37°C in a humidified atmosphere containing 5% CO_2_.

### Measurements of cells’ proliferation rates

Cell imager IncuCyte S3 (Sartorius AG, Göttingen, Germany) measures cell proliferation by live-cell time-lapse imaging, without labels, using Classic Confluence Analysis. IncuCyte S3 live cell analysis system enables real-time, automated quantification of cell proliferation assays inside a tissue culture incubator.

### RNA extraction, cDNA synthesis and real-time qPCR

Total RNA, including miRNAs, from cells was isolated using TRIzol reagent (Invitrogen, Waltham, MA, USA) following manufacturer protocol with overnight RNA precipitation (excluding RNA samples for RNA-seq). Complementary DNA (cDNA) was synthesized from total RNA using qScript cDNA Synthesis Kit (Quanta BioSciences Beverly, MA, USA, cat# 95047–100) for messenger RNA (mRNA) analysis, and using the qScript microRNA cDNA Synthesis Kit (Quanta BioSciences Beverly, MA, USA, cat# 95107–100) for miRNA analysis. Quantitative real-time reverse-transcription polymerase chain reaction (qRT-PCR) of miRNAs and mRNA was performed with the CFX384 Real-Time PCR System (Bio-Rad, Hercules, CA, USA) and a SYBR Green PCR Kit (Quanta BioSciences Beverly, MA, USA, cat# 84018 and 84,071, respectively). The fold expression and statistical significance were calculated using the 2^–ΔΔCt method. All samples from each experiment were tested in triplicates. The primers used for qRT-PCR are shown in Supplementary Table S1. Expression of target miRNAs was normalized to SNORD44 expression, and genes expression – to that of EEF2. Each sample was subjected to minus Reverse Transcriptase control, and miR cDNA – also to minus PolyA Polymerase control.

### Immunoblotting

Protein samples were prepared by lysis cells using protein lysis buffer (Triton × 1%, Tris pH 7.8 10 mm, NaCl 150 mm, NaPP 5 mm, NaF 10 mm, SDS 0.1%, PMSF 1 μM, orthovanadate 4 μM, DTT 1 μM and protease inhibitor cocktail (complete, EDTA free, Roch, Basel, Switzerland)). Protein extracts (25 μg) were resolved on SDS-PAGE (Mini-Protean Tetra System, BioRad, Hercules, CA, USA) and electrophoretically transferred (Trans-Blot Turbo Semi-dry, BioRad, Hercules, CA, USA) onto PVDF membranes (Bio-Rad, Hercules, CA, USA). Chemoluminescent bands were detected by ChemiDoc MP System (BioRad, Hercules, CA, USA) and quantified using ImageJ imaging software (ImageJ, RRID: SCR_003070).

Antibodies used for immunoblotting analyses: anti-CRISPR-Cas9 (ab191468, Abcam, Cambridge, UK), CD47 mAb (clone CC2C6; Biolegend, San Diego, CA, USA), β-actin (Sigma Aldrich, St. Louis, Missouri, USA). Immunoblots were developed with either anti-rabbit or anti-mouse HRP polymer conjugate (Agilent Dako, Santa Clara, CA, USA).

### Generation of Cas9-expressing HCC cell lines

Huh7 and FLC4 cells were transfected with a lentivirus expressing Cas9 protein under the EF1A promoter with hygromycin resistance marker (ID: CAS9HYGROV, Sigma-Aldrich, St. Louis, Missouri, USA). Two days post transfection, cells underwent hygromycin selection for 10 days. Selected cells were seeded in 96 well plates with seeding density 0.5 cells per well. Then, single cell clones were isolated. Protein extracts from selected clones were examined for Cas9 expression by immunoblotting.

### gRNA cloning

gRNAs from the LX-miR library [68] were selected (#25065 targeting miR-222 and the non-targeting negative control_103_GeCKOv2). gRNAs were synthesized by IDT (Coralville, Iowa, United States) with a 5’ phosphate modification at the linker sequence (CACC or AAAC). gRNAs were annealed and ligated into the gRNA expression plasmid (Addgene #112915, Watertown, MA, USA) following the provided protocol [68].

### Virus preparation

Viruses were produced by transient co-transfection of three plasmids into 293T cells as described earlier [69,70] with several modifications. Briefly, 3 × 10^6^ 293T cells were transfected using the Lipofectamine 2000 Transfection Reagent (Thermo Fisher, Waltham, MA, USA) with a total of 20 μg of plasmids DNA: 3.5 μg of the envelope plasmid pMD.G harbouring the gene encoding VSV-G, 6.5 μg of the packaging plasmid pCMVΔR8.91, and 10 μg of the gRNA expression vector. The medium was replaced 20–24 hours after transfection. The medium containing the viral particles was collected 48 and 72 hours after transfection and filtered through 0.45-μm filters (Sartorius, Goettingen, Germany).

### Determining Cas9 cutting efficiency using CD47 marker

Huh7/Cas9 and FLC4/Cas9 positive clones determined by immunoblotting were transduced with either control gRNA or gRNA targeting CD47. After 10 days of blasticidin selection cells were trypsinized and immunostained using APC-conjugated anti-human CD47 mAb (1 µg/ml, clone CC2C6; Biolegend, San Diego, CA) for 30 min on ice. Cells were then washed twice in 0.22 μm filtered PBS, and analysed by FACS. Clones with cutting efficiency >85% were chosen for generation of miR-222-knockout mutants.

### Generation of miR-222-KO and control cells

Huh7/Cas9 and FLC4/Cas9 clones were transduced with either gRNA targeting miR-222 (#25065), or control gRNA (#control_103; sequences – in Supplementary Table S1). The #25065 gRNA is complementary to the last 11 bases of miR-222-5p and to the first 8 bases of the loop between miR-222-5p and miR-222-3p. Thus, all miR-222-KO mutants have mutations in the vicinity of this region. The gRNA vector carries blasticidin resistance gene, thus, cells were selected 48 hours post-transduction with blasticidin for 10 days. The selected cell populations are designated as bulks of miR-222-KO (ko) or control (cg) cells. From these bulk populations single cell clones were isolated and validated for their knockout efficiency by absence of miR-222 expression.

### RNA sequencing

RNA for RNA-seq was purified from single cell clones of Huh7/miR-222-KO and of Huh7/control gRNA (4 clones in each group) using micro miRNeasy kit with an additional DNAse treatment on column (Qiagen, Hilden, Germany ID 217,084 and 79,254). Samples with an RNA integrity number (RIN) >9 were included for further processing. All samples were processed in one batch to minimize batch variations. Stranded RNA-seq (one side, sequencing length: 50–75 bp, 25 million reads per sample) was performed by The Center for Genomic Technologies, The Hebrew University, Jerusalem, Israel. Libraries were prepared using the Lexogen QuantSeq 3’ mRNA-Seq Library Prep Kit FWD (Lexogen, Vienna, Austria), which was followed by an amplification step according to the manufacturer’s instructions. Sequencing was performed on the HiSeq 2500 platform (Illumina, San Diego, USA).

### Bioinformatics

Bioinformatic predictions of miRs’ targets were done using TargetScan (Whitehead Institute for Biomedical Research, Cambridge, MA, USA) and mirDIP (University of Toronto, Toronto, Canada) software and databases.

Bioinformatic analysis of RNA-seq results was performed by Jonathan Monin (Hebrew University of Jerusalem, Israel). The detailed protocol of bioinformatic analysis of the RNA-seq results is described in the Supplementary Methods.

### Retrotransposition assay (RTA)

RTA was performed as described in [29] with some modifications for HCC cell lines and RNA transfections. Briefly, cells were seeded at day 0, and next day (day 1), at 50–60% confluence, were transfected with the RTA system plasmids (pLRE3-EGFP or pJM111-EGFP negative control: 0.35 μg plasmid for 35–45 × 10^3^ cells per well in a 48-well plate) with or without miR-mimics or inhibitors, or siRNAs at 20–60 nM. DharmaFECT Duo (Dharmacon, Lafayette, Colorado, USA) transfection reagent was used for these experiments. Medium was changed twice: at 6 hrs (day 1) and 24 hrs (day 2) post transfection. At day 4, cells were selected with puromycin for episomal expression of plasmids. Selection medium was changed every other day till day 10, when EGFP expression in cells was measured and calculated by Cytoflex FACS (Beckman Coulter, Brea, California, USA) to determine the RTP rate (% of EGFP-positive cells). For RTAs with siRNAs, a pre-treatment with siRNA was performed at 48 hrs before RTA; then, LRE3 + siRNA was added (cells were seeded at day ‘−1’, 1^st^ transfection at day 0 with siRNA only, 2^nd^ transfection with RTA system plasmids and siRNA at day 2). For RTAs with miR mimics and antagos, a second transfection was made at day 3 with miR mimics or antagos only (cells were seeded at day 0, 1^st^ transfection with RTA system plasmids and miR mimics or antagos at day 1, then 2^nd^ transfection at day 3 with miR inhibitor/mimic only).

### Global proteomics

Bulks of miR-222-KO and control gRNA-expressing Huh7 and FLC4 cells were grown to 60–70% confluence, then cells were trypsinized and washed three times with PBS. About 10^6 cells were pelleted and kept at −80°C until sending them for proteomics analysis. Global proteomics analysis was performed by two specialized centres: the Smoler Proteomics Center at the Technion, Haifa, Israel (two rounds), and the Stein Family Mass Spectrometry Center, The Silberman Institute of Life Sciences, Hebrew University of Jerusalem, Israel (one round). The detailed protocols of global proteomics analyses are described in the Supplementary Methods.

### Statistical analysis

Data were evaluated for significance by one way ANOVA or two-tailed Student t-test or Mann – Whitney test, as indicated and unless otherwise noted. P-value: *, *p* ≤ 0.05; **, *p* < 0.001; ****, *p* < 0.0001. Calculations were performed using GraphPad Prism 6.02 software (GraphPad Software, Boston, MA, USA).

List of oligos and plasmids – in Supplementary Table S1.

## Supplementary Material

Supplementary_information_corrected.docx

## Data Availability

RNA-seq data is accessible in GEO: GSE273369. Any further underlying data will be made available upon reasonable request from the corresponding author.
