# Supplementary material for: The oncogenic microRNA miR-222 promotes human LINE-1 retrotransposition
Source: RNA Biol. 2025 May 27;22(1):1–15. doi: 10.1080/15476286.2025.2511318 (PMC12143727; doi:10.1080/15476286.2025.2511318)
Supplement: Supplementary_information_corrected.docx [file KRNB_A_2511318_SM6430.docx]

**Supplementary information**

**Supplementary Table 1 Oligonucleotides and plasmids used in this study.**

| **Oligo RNAs** | **Catalog #** | **Sequence** | **Comments** |
| --- | --- | --- | --- |
| **Sigma Aldrich** |  |  |  |
| antago-control MISSION® Synthetic microRNA Inhibitor | NCSTUD001 | A double-stranded RNA molecule designed to inhibit the control sequence: GGUUCGUACGUACACUGUUCA | ath-miR416, Negative Control 1, Sequence from *Arabidopsis thaliana* with no homology to human and mouse gene sequences |
| antago-miR-221-3p MISSION® Synthetic microRNA Inhibitor | hstud0398 | A double-stranded RNA molecule designed to inhibit the mature miR-221-3p: AGCUACAUUGUCUGCUGGGUUUC |  |
| antago-miR-222-3p MISSION® Synthetic microRNA Inhibitor | hstud0400 | A double-stranded RNA molecule designed to inhibit the mature miR-222-3p: AGCUACAUCUGGCUACUGGGU |  |
| **IDT** |  |  |  |
| **antagos** |  |  |  |
| NC5 AMO antagomir |  | mG/ZEN/mC mGmAmC mUmAmU mAmCmG mCmGmC mAmAmU mAmUmG mG/3ZEN/ |  |
| miR-222-3p antagomir |  | mA/ZEN/mC mCmCmA mGmUmA mGmCmC mAmGmA mUmGmU mAmGmC /3ZEN/ |  |
| mir-99a-5p antagomir |  | mC/ZEN/mA mCmAmA mGmAmU mCmGmG mAmUmC mUmAmC mGmGmG mU/3ZEN/ |  |
| let-7c-5p antagomir |  | mA/ZEN/mA mCmCmA mUmAmC mAmAmC mCmUmA mCmUmA mCmCmU mC/3ZEN/ |  |
| mir-125b-5p antagomir |  | mU/ZEN/mCmAmCmAmAmGmUmUmAmGmGmGmUmCmUmCmAmGmGmG/3ZEN/ |  |
| **mimic miRs** |  |  |  |
| NC5 mimic |  | Mature strand: /5Phos/rGrC rGrArC rUrArU rArCrG rCrGrC rArArU rArUmG mGrU Complimentary strand: mCmArU mArUmU rGmCrG mCrGmU rAmUrA mGrUmC rGC |  |
| miR-222-3p mimic |  | Mature strand: /5PHOS/rArGrCrUrArCrArUrCrUrGrGrCrUrArCrUrGmGmGrU Complimentary strand: mCmCrAmGrUmArGmCrCmArGmArUmGrUmArGmCU |  |
| miR-222-5p mimic |  | Mature strand: /5Phos/rCrU rCrArG rUrArG rCrCrA rGrUrG rUrArG rArUmC mCrU Complimentary strand: mGmArU mCrUmA rCmArC mUrGmG rCmUrA mCrUmG rAG |  |
| **siRNAs** |  |  |  |
| miR222HG-1 (mix1) |  | rGrArArUrGrArCrArGrArGrArArCrUrUrGrUrArArArGrATA and rUrArUrCrUrUrUrArCrArArGrUrUrCrUrCrUrGrUrCrArUrUrCrCrA |  |
| miR222HG-2 (mix1) |  | rArGrArUrUrGrArCrArArCrUrUrArCrArUrGrUrUrGrUrUTA and rUrArArArCrArArCrArUrGrUrArArGrUrUrGrUrCrArArUrCrUrUrU |  |
| miR222HG-3 (mix2) |  | rUrCrUrGrArArUrCrArCrUrCrArGrGrArArUrUrCrArArGCA and rUrGrCrUrUrGrArArUrUrCrCrUrGrArGrUrGrArUrUrCrArGrArUrU |  |
| miR222HG-4 (mix2) |  | rCrArUrArCrUrGrCrCrUrUrArCrArArCrCrUrUrArUrUrCCT and rArGrGrArArUrArArGrGrUrUrGrUrArArGrGrCrArGrUrArUrGrArU |  |
| Negative Non-targeting or scrambled Control DsiRNA by IDT | 51-01-14-04 NC1   51-01-19-09- scrambled |  | siRNA controls with no homology to human gene sequences |
| **gRNAs** |  |  |  |
| miR-222-25065 |  | GCGAAAGACAGGATCTACAC | #25065 in the Lx-miR gRNA library |
| control_103_  GeCKOv2 |  | GACCCTCCGGATACGGACTG | #Control_103 in the Lx-miR gRNA library |
| **Primers** |  |  |  |
| MIR222HG-F1 |  | CTCCCTGCCTCAACTGTCAA |  |
| MIR222HG-R1 |  | AGGCTGGTGTGGTAAAGGGA |  |
| MIR222HG R2 |  | CGGTCCTTTCTCTGCACTC |  |
| lINC02595 F2 |  | TGGCAGTGTAGAAGCCAGAC |  |
| miR-222-3p |  | AGCTACATCTGGCTACTGGGT |  |
| miR-222-5p |  | CTCAGTAGCCAGTGTAGATCCT |  |
| miR-99a-5p |  | AACCCGTAGATCCGATCTTGTG |  |
| let-7c-5p |  | TGAGGTAGTAGGTTGTATGGTT |  |
| miR-125b-5p |  | TCCCTGAGACCCTAACTTGTGA |  |
| **Plasmids** |  |  |  |
| pLRE3-EGFP | LINE-1 element with EGFP reporter disrupted by inverted intron and puromycin selection | Kindly provided by John V. Moran (Department of Human Genetics, University of Michigan, Ann Arbor, MI, USA) |  |
| pJM111-LRE3-EGFP | Mutated LINE-1 element with EGFP reporter disrupted by inverted intron and puromycin selection | Kindly provided by John V. Moran (Department of Human Genetics, University of Michigan, Ann Arbor, MI, USA) |  |
| pJM101/L1.3 | LINE-1 element with NEO reporter disrupted by inverted intron and hygromycin selection | Kindly provided by John V. Moran (Department of Human Genetics, University of Michigan, Ann Arbor, MI, USA) |  |
| pJm111/L1.3 | LINE-1 element with NEO reporter disrupted by inverted intron and hygromycin selection | Kindly provided by John V. Moran (Department of Human Genetics, University of Michigan, Ann Arbor, MI, USA) |  |
| pEGFP-N3  (Clontech) (EGFP and neomycin resistance) | Control for clonability assay |  |  |
| gRNA expression pLX-sgRNA | Addgene #112915 |  |  |
| pLX-miR pooled gRNA library | Addgene #112200 |  |  |

**Supplementary materials and methods**

**RNA-seq analysis: differentially expressed genes and GSEA**

Quality assessment measures were first applied to FASTQ files with FastQC v0.11.8 tool (http://www.bioinformatics.babraham.ac.uk/projects/fastqc).

Reads were next aligned to the transcriptome (HSA GRCh38) using Salmon v1.10.0, a tool for fast and bias-aware quantification of transcript expression (Patro, R., Duggal, G., Love, M. I., Irizarry, R. A., & Kingsford, C. 2017, Nature Methods). For this mapping the tag gcBias was enabled.

Principle Component analysis and clustering were performed with native R (http://www.R-project.org/) functions and custom R scripts.

Differential gene expressions were explored with DESeq2, (Love, M.I., Huber, W., Anders, S. Moderated estimation of fold change and dispersion for RNA-seq data with DESeq2 Genome Biology 15(12):550, 2014). Fold change was calculated with apeglm V1.22.1 method (Zhu, A., Ibrahim, J.G., Love, M.I. Heavy-tailed prior distributions for sequence count data: removing the noise and preserving large differences. Bioinformatics 2018).

Over representation analysis was performed with enrichR v3.2 (Enrichr: interactive and collaborative HTML5 gene list enrichment analysis tool, Edward Y Chen 1, Christopher M Tan, Yan Kou, Qiaonan Duan, Zichen Wang, Gabriela Vaz Meirelles, Neil R Clark, Avi Ma'ayan, PMID: 23586463 PMCID: PMC3637064 DOI: 10.1186/1471-2105-14-128). The top 500 most significant genes were tested for over representation.

Gene Set Enrichment Analysis for pathways and GO terms was performed with Broad Institute GSEA utility V4.2.1 (GSEA software, and Molecular Signature Database (MSigDB), Subramanian, Tamayo, et al. (2005), PNAS 102, 15545-15550, http://www.broad.mit.edu/gsea). For this analysis, genes were ranked by a signed p-value adopted from the DEG step.

Heatmaps were created with R package gplots V3.1.3 utility.

**Global proteomics (Smoler Proteomics Center at the Technion (Haifa, Israel)**

**Proteolysis**

Cells were lyzed in 8.5 M Urea, 400 mM ammonium bicarbonate and 10 mM DTT, sonicated twice (90%, 10-10, 5’), and centrifuged (10,000g, 10’). Protein amount was estimated using Bradford readings. The samples were reduced (60ºC for 30 min), modified with 35.2 mM iodoacetamide in 100 mM ammonium bicarbonate (room temperature for 30 min in the dark) and digested in 1.5 M Urea, 66 mM ammonium bicarbonate with modified trypsin (Promega), overnight at 37^o^C in a 1:50 (M/M) enzyme-to-substrate ratio. An additional second digestion with Trypsin was done for 4 hours at 37oC in a 1:100 (M/M) enzyme-to-substrate ratio. The tryptic peptides were desalted using homemade C18 stage tip, dried and re-suspended in 0.1% Formic acid.

**Mass spectrometry analysis**

The resulted peptides were analyzed by LC-MS/MS using a Q Exactive HFX or Exploris 480 mass spectrometer (Thermo) fitted with a capillary HPLC (Ultimate 3000, Thermo Scientific, or Easy nLC 1200, respectively).

Exploris 480 mass spectrometer: The peptides were loaded in solvent A (0.1% formic acid in water) on a homemade capillary column (30 cm, 75-micron ID) packed with Reprosil C18-Aqua (Dr. Maisch GmbH, Germany).

The peptides mixture was resolved with a 6 to 34% linear gradient of solvent B (80% acetonitrile with 0.1% formic acid) for 180 minutes followed by gradient of 15 minutes of 34 to 80% and 15 minutes at 80% acetonitrile with 0.1% formic acid in water at flow rates of 0.15 μl/min. Mass spectrometry was performed by in a positive mode (m/z 350-1200, resolution 120,000 for MS1 and 15,000 for MS2) using repetitively full MS scan followed by high collision dissociation (HCD, at 27 normalized collision energy) of the 30 most dominant ions (>1 charges) selected from the first MS scan. The AGC settings were 3x106 for the full MS and 1x105 for the MS/MS scans. The intensity threshold for triggering MS/MS analysis was 1x104. A dynamic exclusion list was enabled with an exclusion duration of 30 s.

**Data analysis**

The mass spectrometry data was analyzed using Protein Discoverer 2.4 (Thermo) using Sequest search engine, searching against the Human proteome UP000005640 from the Uniprot database (downloaded in July 2021, [78,139](https://www.uniprot.org/uniprotkb?query=proteome:UP000005640) entries) with mass tolerance of 20 ppm for the precursor masses and 0.02 Da for the fragment ions. Oxidation on methionine, and protein N-terminus acetylation were accepted as variable modifications and carbamidomethyl on cysteine was accepted as static modifications. Minimal peptide length was set to six amino acids and a maximum of two miscleavages was allowed. The data was quantified by label free analysis using the same software. Peptide-level false discovery rates (FDRs) were filtered to 1% using the target-decoy strategy.

**Global proteomics (the Stein Family Mass Spectrometry Center, The Silberman Institute of Life Sciences, The Hebrew University, Jerusalem, Israel)**

**Sample preparation**

Cells were lysed in 25 mM Tris-HCl pH 8.0 containing 5% sodium dodecyl sulfate, heated at 95oC for 5 min. and sonicated in a bath sonicator to shear DNA. The proteins were reduced by the addition of 10 mM dithiothreitol for 30 min and alkylated by addition of 55 mM iodoacetamide (Sigma Chem. Corp. St. Louis, MO) and incubation for 30 min. at room temperature in the dark. Removal of SDS followed by digestion with sequencing grade modified trypsin (Promega Corp., Madison, WS) were performed using the S-Trap microspin column kit as specified by the manufacturer (Protifi, LLC, Huntington, NY). The tryptic peptides were desalted on home-made C18 Stage tips. A total of 0.3 µg of peptides (determined by Absorbance at 280 nm) from each sample were injected into the mass spectrometer.

**Nano-LC-MS/MS analysis**

MS analysis was performed using a Q Exactive-HF mass spectrometer (Thermo Fisher Scientific, Waltham, MA USA) coupled on-line to a nanoflow UHPLC instrument, Ultimate 3000 Dionex (Thermo Fisher Scientific, Waltham, MA USA). Peptides dissolved in 0.1% formic acid were separated without a trap column over an 120 min acetonitrile gradient run at a flow rate of 0.3 μl/min on a reverse phase 25-cm-long C18 column (75 μm ID, 2 μm, 100Å, Thermo PepMapRSLC). The instrument settings were as described by Scheltema et al. (Scheltema RA, Hauschild JP, Lange O, Hornburg D, Denisov E, Damoc E, Kuehn A, Makarov A, Mann M. Mol Cell Proteomics. 2014 Dec;13(12):3698-708. The Q Exactive HF, a Benchtop mass spectrometer with a pre-filter, high-performance quadrupole and an ultra-high-field Orbitrap analyzer.) Survey scans (300–1,650 m/z, target value 3E6 charges, maximum ion injection time 20 ms) were acquired and followed by higher energy collisional dissociation (HCD) based fragmentation (normalized collision energy 27). A resolution of 60,000 was used for survey scans and up to 15 dynamically chosen most abundant precursor ions, with “peptide preferable” profile were fragmented (isolation window 1.6 m/z). The MS/MS scans were acquired at a resolution of 15,000 (target value 1E5 charges, maximum ion injection times 25 ms). Dynamic exclusion was 20 sec. Data were acquired using Xcalibur software (Thermo Scientific). To avoid a carryover, the column was washed with 80% acetonitrile, 0.1% formic acid for 25 min between samples.

**MS data analysis**

Mass spectra data were processed using the MaxQuant computational platform, version 2.0.3.0. Peak lists were searched against human proteome database UP000005640 from Uniprot. The search included cysteine carbamidomethylation as a fixed modification, N-terminal acetylation and oxidation of methionine as variable modifications and allowed up to two miscleavages. The ‘match-between-runs’ option was used. Peptides with a length of at least seven amino-acids were considered and the required FDR was set to 1% at the peptide and protein level. Relative protein quantification in MaxQuant was performed using the label-free quantification (LFQ) algorithm (Cox, J. et al. MaxLFQ allows accurate proteome-wide label-free quantification by delayed normalization and maximal peptide ratio extraction. Mol. Cell. Proteomics 13, 2513–2526 (2014).

Statistical analysis (*n=4*) was performed using the Perseus statistical package (Tyanova S, Temu T, Sinitcyn P, Carlson A, Hein MY, Geiger T, Mann M, Cox J. Nat Methods. 2016 Sep; 13(9):731-40. doi: 10.1038/nmeth.3901. PMID: 27348712. The Perseus computational platform for comprehensive analysis of (prote)omics data). Only those proteins for which at least 3 valid LFQ values were obtained in at least one sample group were accepted for statistical analysis by Volcano plot (t-test, *p* < 0.05). After application of this filter, a random LFQ value was substituted for proteins for which LFQ could not be determined ("Imputation" function of Perseus). The imputed values were in the low range of the median value of all the proteins in the sample and allowed calculation of p-values.

**Legends to supplementary figures**

**Supplementary Figure 1. Kaplan-Meier plots of miR-221-3p and miR-222-3p in HCC.** Kaplan-Meier plots of miR-221-3p

(**A**, designated as miR-221) and miR-222-3p (**B**, designated as miR-222) in 372 HCC patients, using accessible public databases

(Posta, M. & Gyorffy, B. Analysis of a large cohort of pancreatic cancer transcriptomic profiles to reveal the strongest prognostic factors.

*Clin Transl Sci* **16**, 1479-1491, doi:10.1111/cts.13563 (2023)).

**Supplementary Figure 2. CD47 staining demonstrates cutting efficiency (CE) in clones of HCC cell lines stably expressing Cas9 protein.** **A, B** CD47 protein level in Huh7/Cas9 cells without (**A**) or with (**B**) gRNA targeting CD47 (85.73% CE). **C, D** CD47 protein level in FLC4/Cas9 cells without (**C**) or with (**D**) gRNA targeting CD47 (95.73% CE). Single-cell-derived Cas9-expressing clones were analyzed 10 days following transduction with a lenti-vector expressing gRNA targeting CD47.

**Supplementary Figure 3. Loss of miR-222 does not affect cell proliferation rate in Huh7 and FLC4 cells.** Proliferation rate assessment

through live, label-free cell imaging of Huh7 (**A**) and FLC4 (**B**) cells performed by the IncuCyte S3 system. The 7-day experiment demonstrates

comparable proliferation rates between Cas9-expressing miR-222 knockout (highlighted in red) and control (highlighted in green) cells, as well

as parental cells without Cas9 (depicted in black). Cell confluence measurements at specific time points were normalized to the initial confluence, providing insights into the relative growth dynamics over the experimental time.

**Supplementary Figure 4. Expression of miR-222/221-3p & -5p arms in miR-222 KO cells.** Mir222/221 arms expression in Huh7 (**A**) and FLC4 (**B**) cells stably expressing Cas9 and either control gRNA or gRNA to miR-222 (miR-222-KO cells). **C, D** melting curves for miR-222-3p & 5p qPCR-amplified products in FLC4 cells harboring either miR-222-KO or control gRNA (similar results in Huh7). It can be seen that melting curve for miR-222-5p in miR-222-KO cells differs from that in cells expressing control gRNA, demonstrating that the qPCR product in this case is not produced by miR-222-5p, and thus, miR-222-KO cells do not express miR-222-5p. **E, F** melting curves for mir-221-3p & 5p qPCR-amplified products in FLC4 and Huh7 cells harboring either miR-222KO or control gRNA (all cells used in these experiments were bulk miR-222-KO or control gRNA expressing cells). * p<0.05, ** p<0.005.

**Supplementary Figure 5. The most prominent enrichment plots produced by GSEA of RNA-seq data of stable**

**Huh7/miR-222-KO vs. Huh7/control-gRNA clones (4 clones in each group; C2 refers to a set of several databases).**

**Supplementary Figure 6. Validation by RT-PCR of upregulation of the known MIR222HG transcript and the new spliced LINC02595- MIR222HG transcript in the absence and following knockdown of miR-222-3p. A** Increased expression of the MIR222HG transcript in stable Huh7 miR-222-KO mutants versus Huh7 stable cells expressing control gRNA (4 single cell clones in each group; RT-PCR with primers F1, R1). **B** Transcripts encoded in the MIR222HG – LINC02595 region of human chromosome X (as presented by Ensembl). **C** The spliced LINC02595 - MIR222HG chimeric transcript is amplified only in the miR-222-KO derivatives of Huh7 and FLC4 cell lines (RT-PCR with primers F2, R2; “-RT” controls were negative for both EEF2 and LINC02595 - MIR222HG chimeric transcript in all samples). **D** The spliced

LINC02595 - MIR222HG chimeric transcript is amplified only in HeLa, Huh7 and FLC4 cells treated with antago-miR-222-3p, but not following treatment with the control antago-mir. **C, D** NTC – no template control. Lower panels – RT-PCR of the housekeeping EEF2 gene. **E** Spliced variant exons predicted by RNA-seq read annotation for the chimeric transcript of LINC02595 and MIR222HG. Black arrows represent primers for the 340 bp PCR product. The PCR products were isolated from gel and sent for sequencing. **F** Expected sequence of the spliced LINC02595 - MIR222HG chimeric transcript derived from alignments of the 75 bp RNA-seq reads of Huh7 miR-222-KO cells. Color codes: blue and yellow – two last exons of LINC02595, cyan, green and orange – three exons of MIR222HG. Sequence between primers F2 and R2 was determined from RNA-seq results and sequencing of the appropriate PCR-products; sequences in blue and orange – reconstructed from the IGV files of RNA-seq results. **G** Sequence results validated the presence of the new chimeric transcript in all three cell lines treated with antago-miR-222-3p (sequence results for FLC4/miR-222-KO cells were identical

to those shown for FLC4 cells transfected with antago-miR-222-3p).

**Supplementary Figure 7. Clusters of tumor suppressor miRs 99/100-let-7-125 in human genome.** Three genomic regions, each encoding three tumor suppressor miRs: members of the 99/100, let-7 and 125 families. For each family, -5p arms of miRs in all three regions are highly homologous and share the same seed sequence (125b1 and 125b2 are completely identical); their -3p arms are less homologous and have different seed sequences.

**Supplementary Figure 8. Antago-let-7c rescues LINE-1 RTP in Huh7/miR-222-KO cells in vitro.** RTA in Huh7/miR-222-KO cells (bulk)

using LRE3-EGFP vector alone or together with antago-miRs (a-miRs): a-miR-99a-5p and a-miR-let-7c-5p, or control a-ctrl (JM111 – negative

control retrotransposition-defective mutant of LRE3). Concentration of antago-miRs: 40 nM for each one when only one type is applied;

20 nM for each one when two antago-miRs are applied together (40 nM total); ** p<0.005.

**Supplementary Figure 9. Proteomic analysis of Huh7 and FLC4 miR-222-KO cells.** Numbers of proteins differentially expressed in miR-222-KO (ko) versus control gRNA-expressing (cg) Huh7 or FLC4 cells (three global proteomics rounds for each cell type; fold-change threshold: 1.8x). Data in red and blue – two rounds performed at the Israeli Technological Institute, data in black – the third round performed at The Hebrew University. There were only three common differentially expressed proteins in three rounds of these global proteomics experiments: COL2A1 protein was downregulated in Huh7cells, while BAAT and METTL7A proteins were upregulated in FLC4 cells.

**Supplementary Figure 10. Kaplan-Meier plots of miR-99a-5p, let-7c-5p and miR-125-5p in HCC.** Kaplan-Meier plots of miR-99a-5p

(**A**, designated as miR-99a), let-7c-5p (**B**, designated as let-7c) and miR-125b-5p (**C**, designated as miR-125b) in 372 HCC patients, using

accessible public databases (Posta, M. & Gyorffy, B. Analysis of a large cohort of pancreatic cancer transcriptomic profiles to reveal the

strongest prognostic factors. *Clin Transl Sci* **16**, 1479-1491, doi:10.1111/cts.13563 (2023)).

**Supplementary Figure 11. Loss of miR-222 does not change significantly the steady-state level of LINE-1-EGFP RNA following cell transfection with pLRE3-EGFP plasmid.** Huh7 (A) or FLC4 (B) cells were transfected with plasmid pLRE3-EGFP (that was used in this study for LINE-1 RTA), and, two days later, total RNA was isolated. RT-PCR was performed using primers targeting LRE3 ORF2 (forward) and EGFP (reverse), as well as primers for puromycin resistance transcript for normalization to plasmid, and primers to EEF2 gene for normalization

to cells numbers. Experiment was done with quadruplicates of bulks of miR-222-KO cells (ko 1-4) and bulks of control gRNA cells (cg 1-4).

**Supplementary Figure 12. Loss of miR-222 does not change significantly the endogenous level of miR-128-3p in Huh7 cells.**

Endogenous levels of miR-128-3p in single clones of Huh7/miR-222-KO and Huh7/control gRNA- expressing cells (four clones in each group).
